# Refractive Errors in 3–6 Year-Old Chinese Children: A Very Low Prevalence of Myopia?

**DOI:** 10.1371/journal.pone.0078003

**Published:** 2013-10-30

**Authors:** Weizhong Lan, Feng Zhao, Lixia Lin, Zhen Li, Junwen Zeng, Zhikuan Yang, Ian G. Morgan

**Affiliations:** 1 State Key Laboratory of Ophthalmology, Zhongshan Ophthalmic Center, Sun Yat-sen University, Guangzhou, Guangdong, China; 2 Shanxi Eye Hospital, Taiyuan, Shanxi, China; 3 Aier Eye Hospital Group, Changsha, Hunan, China; 4 ARC Centre of Excellence in Vision Science, Research School of Biology, Australian National University, Canberra, Australia; 5 Department of Preventive Ophthalmology, State Key Laboratory of Ophthalmology, Zhongshan Ophthalmic Center, Sun Yat-sen University, Guangzhou, Guangdong, China; Zhongshan Ophthalmic Center, China

## Abstract

**Purpose:**

To examine the prevalence of refractive errors in children aged 3–6 years in China.

**Methods:**

Children were recruited for a trial of a home-based amblyopia screening kit in Guangzhou preschools, during which cycloplegic refractions were measured in both eyes of 2480 children. Cycloplegic refraction (from 3 to 4 drops of 1% cyclopentolate to ensure abolition of the light reflex) was measured by both autorefraction and retinoscopy. Refractive errors were defined as followed: myopia (at least −0.50 D in the worse eye), hyperopia (at least +2.00 D in the worse eye) and astigmatism (at least 1.50 D in the worse eye). Different definitions, as specified in the text, were also used to facilitate comparison with other studies.

**Results:**

The mean spherical equivalent refractive error was at least +1.22 D for all ages and both genders. The prevalence of myopia for any definition at any age was at most 2.5%, and lower in most cases. In contrast, the prevalence of hyperopia was generally over 20%, and declined slightly with age. The prevalence of astigmatism was between 6% and 11%. There was very little change in refractive error with age over this age range.

**Conclusions:**

Previous reports of less hyperopic mean spherical equivalent refractive error, and more myopia and less hyperopia in children of this age may be due to problems with achieving adequate cycloplegia in children with dark irises. Using up to 4 drops of 1% cyclopentolate may be necessary to accurately measure refractive error in paediatric studies of such children. Our results suggest that children from all ethnic groups may follow a similar pattern of early refractive development, with little myopia and a hyperopic mean spherical equivalent over +1.00 D up to the age of 5–6 yearsin most conditions.

## Introduction

The conventional picture of neonatal refractive development is derived from small studies on children of predominantly European ancestry in Europe [1]–[3] and the United States [4]–[6]. It suggests that children are born with a normal distribution of refractive error, with a significantly hyperopic mean refractive error. Over the first year or two after birth, this distribution is sharpened by a reduction in the relatively rare myopic refractive errors, and a reduction in hyperopic errors, leading to a narrower distribution of spherical equivalent refractive error (SE) characterised by significant kurtosis, but with the peak in the hyperopic, rather than emmetropic, range. At this age, the prevalence of myopia is very low, of the order of 1–2% at most. These changes in distribution appear to result from a process in which the axial length (AL) of the eye is matched to the corneal radius of curvature (CR), since by the age of 5–6, the ratio of the AL to the CR, in addition to SE, shows increased kurtosis7. In contrast, other biometric parameters show essentially normal distributions.

Few studies have followed the continuing development of these characteristics through childhood and into the adult years, but by the age of 5–7, these characteristics of a hyperopic mean SE and a tight distribution of SE have been reported in most populations which have been studied [7]–[16]. In populations where the prevalence of myopia is low, these characteristics are preserved in adult populations [17], whereas in populations where the prevalence of myopia has become high, such as in major cities in China, the mean SE becomes myopic and the kurtosis in the distribution is lost [11].

Recently, some large-scale population-based studies of paediatric populations, specifically the Multi-Ethnic Pediatric Eye Disease Study (MEPEDS) of children of African-American and Hispanic ethnicity in Los Angeles [18] the Baltimore Pediatric Eye Disease Study (BPEDS) of white and African-American children in Baltimore [19], and the Strabismus, Amblyopia and Refraction Study (STARS) from Singapore [20] have reported a somewhat different picture. Very recently the MEPEDS group has also reported on Asian (predominantly Chinese) and non-Hispanic White children from Los Angeles [21]. While the results on white children from both MEPEDS and BPEDS are quite similar to the conventional picture, the studies on other ethnic groups have all reported significant myopia prevalence rates between the ages of 6–12 months and 5–6 years of age. For example, with a cut-off of ≤−1.00 D in three year-olds, in BPEDS, the prevalence of myopia was 0% and the mean SE was +1.25 D in white children, compared to 0.66% and +1.47 D in MEPEDS. The corresponding figures for African-American children were 10.5% and +0.68 D in BPEDS and 5.5% and +1.10 D in MEPEDS, as compared to 1.5% and 1.36 D in children of Hispanic ethnicity in MEPEDS. In STARS, the prevalence of myopia was reported to be 8.6% and the mean SE +0.61 D at this age These data pose an important question, since one interpretation of them is that there is a quite different pattern of refractive development in children from ethnic backgrounds other than white, compared to that which has been described for children of European ancestry.

An alternative explanation could lie in the well-known difficulties associated with achieving complete cycloplegia in children with dark irises [22]. This difficulty was flagged in the MEPEDS, BPEDS and STARS analyses, but has not been critically analysed further. In addition to this problem, the methods of measuring refractive error varied with age in the STARS analysis, further complicating the interpretation. In these paediatric studies, the Retinomax hand-held autorefractor was used for many, if not all, measurements, which may result in some over-estimation of myopia [22]–[24]. Exclusion of the Retinomax measurements reduced the prevalence of myopia in three year-olds in STARS, where the greatest variation in methodology occurred, from 8.6% to 5.9%. This made an even bigger difference for the two year-olds, where the prevalence of myopia dropped from 20.2% to 9.9% after exclusion of the Retinomax data.

In the course of evaluating a kit for home-based screening for amblyopia in Guangzhou [25], we have measured cycloplegic refractive errors, using both retinoscopy and autorefraction, for a large sample of 3–6 year-old children attending pre-schools. We have now analysed our data to see if they confirm the patterns of refractive development reported for children of Chinese ethnicity from Singapore. A preliminary account of some of these data has been published in Chinese [26].

## Materials and Methods

### Ethics Statement

The study was approved by the Ethics Committee of Zhongshan Ophthalmic Center, Guangzhou, China. All study procedures adhered to the tenets of the Declaration of Helsinki. The purpose and methods of the study, including rare complications of cyclopentolate eyedrops, were explained to the parents before written informed consent was obtained.

### Subjects

From February to May, 2009, we completed a kindergarten-based study, testing the validity and cost-effectiveness of a home-based screening kit for amblyopia. The study included children aged 3–6 years, who were from 10 kindergartens randomly selected from Guangzhou, China. The geographical characteristics of the city and the selection methodology have been reported in detail elsewhere [25]. Briefly, Guangzhou consists of 10 administrative districts. The residents are overwhelmingly of Chinese (Han) ethnicity. Four administrative districts, including two central districts, one suburban district, and one more rural district, were randomly identified for the study, based on the populations of these entities. The number of kindergartens in these districts was 125, 155, 219 and 319, respectively. Then one, two, three, and four kindergartens from these four districts were randomly chosen, in order to give approximately the same number of children from each district. The enrolment rate in preschools in Guangzhou is now over 80% (http://www.gzstats.gov.cn/pchb/dwcrk/200903/t20090313_7376.htm).

### Determination of Refractive Error

The eye examinations included an extensive ocular health evaluation, tests of ocular alignment, ocular motility and visual acuity measurements, which have been described elsewhere, and measurement of refractive errors. Refractive error was measured after cycloplegia, using both a table-mounted autorefractor (Topcon AR 8800, Tokyo, Japan) and by retinoscopy. Cycloplegia was induced with two drops of 1% cyclopentolate (Cyclogyl, Alcon, Belgium) instilled five minutes apart, with a third drop administered after 20 minutes. Cycloplegia was then evaluated after an additional 15 minutes. Cycloplegia was considered complete if a light reflex was absent. If a light reflex was still detected, which was the case in 8–10% of children, another drop of cyclopentolate was administered, the light reflex tested after 15 minutes and then refractive errors were measured.

### Data Management and Analysis

Refractive error was expressed as the spherical equivalent (SE, i.e. spherical error plus half of the cylinder error). Myopia was defined as SE of at least −0.50 D, and high myopia as at least −6.00 D. Hyperopia was defined as SE of at least +2.00 D. In order to facilitate comparison of the data with other studies, different definitions were also used for particular analyses, as specified in the text. Astigmatism was defined as a cylindrical measurement of at least 1.50 D and was classified into three categories: with-the-rule astigmatism (cylinder axis between 1° and 15° or 165° and 180°), against-the-rule-astigmatism (cylinder axis between 75° and 105°), and oblique astigmatism (cylinder axis between 16° and 74° or 106° and 164°). In order to facilitate comparison with other studies, the prevalence of refractive errors was reported either for the worse eye or the right eye, as specified in the text.

Statistical analyses were performed using SPSS 16.0 (SPSS Inc., Chicago, IL). The cut-off for statistical significance was set at p<0.05. One-way ANOVA and Chi-square tests were used to analyze the difference among age groups for the mean SE and the prevalence of different types of refractive errors, respectively. 95% confidence intervals (CI) were calculated using the normal approximation or Poisson distribution where appropriate.

## Results

A total of 3,300 children in the randomly selected 10 kindergartens were invited to participate in the study, representing approximately 1% of the total population of 3–6 year-old children and 0.7% of the kindergartens in Guangzhou. Home-based amblyopia screening kits [25] were distributed to the parents and 2,442 self-test reports were returned. An extra 38 parents, who did not successfully complete the home-screening, asked for ocular examinations for their children. Therefore, a total of 2,480 children (75.1% participation) had cycloplegic refraction measurements, including 1,310 (52.8%) boys and 1,170 (47.2%) girls (Table 1). Of the 2,480 children who were examined, refraction data for both autorefraction and retinoscopy was missing in two cases. Thus, data from a total of 2,478 children are presented in this report.

**Table 1:** Age and Gender of the Participants.

Group (yrs)	N	Age (months)		N (% boys)
		Median	IqR	
3	373	44.0	41.0–45.5	201 (53.9)
4	818	54.0	51.0–57.0	434 (53.1)
5	847	65.0	62.0–68.0	437 (51.6)
6	442	75	73.0–77.0	238 (53.8)
All	2480	60.0	52.0–69.0	1310 (52.8)

IqR, interquartile range.

### Measurement Agreement

**Figure 1:**
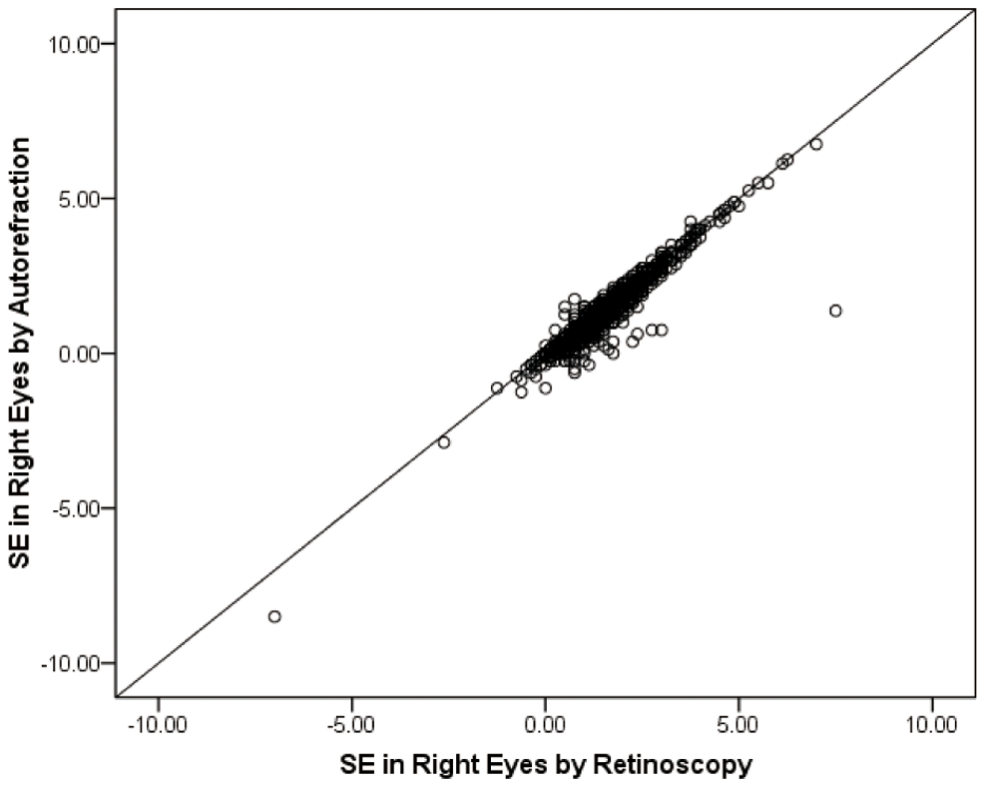
Comparison of refractions measured by autorefraction and retinoscopy in right eyes.

With cycloplegia, retinoscopy and autorefraction measurements were found to be highly correlated (Pearson correlation of 0.949 for right and 0.955 for left eyes, both *P*<0.001), as shown in Figure 1 for right eyes. However, autorefraction gave systematically slightly more negative (less positive) results than retinoscopy. The mean difference was −0.13±0.25 D for right eyes and −0.15±0.24 D for left eyes. Both differences were statistically significant, but are of little clinical significance (Paired tests, *P*<0.001).

### Spherical Equivalent Refractive Error

As the within-subject SE of the eyes correlated highly (Spearman correlation coefficient = 0.88, *P*<0.001 for autorefraction and Spearman correlation coefficient = 0.89, *P*<0.001 for retinoscopy), only data for right eyes are presented for mean SE (Table 2). The mean SE for all the children was +1.42 D (SD: 0.79) and the range was −8.50 D to +6.75 D. Significant differences by age for mean SE were found in the total sample (*P* = 0.03), with this finding being observed in boys (*P*<0.001), but not in girls (*P* = 0.76). Despite the significant age differences detected by ANOVA, there were no clear trends in mean SE with age. There was a significant difference in mean SE between boys (mean SE +1.35 D, SD: 0.71) and girls (mean SE +1.49 D, SD: 0.87; *P*<0.001). A more hyperopic mean SE in girls was observed in all age groups (all *P*<0.01), except in the four year-olds (*P* = 0.70). However, all these differences are too small to be of clinical significance. Similar results were obtained with retinoscopy.

**Table 2:** Spherical Equivalent (SE) in Right Eyes Determined by Autorefraction and Retinoscopy.

Age (yrs)	N	SE (D)					
		Mean	SD	Median	Range	Kurtosis	Skewness
**All children**	**2478**	**1.42(1.54)**	**0.79(0.76)**	**1.38(1.50)**	**15.25(14.50)** *	**13.61(10.78)** #	**0.05(0.39)**
3	373	1.44(1.58)	0.76(0.713)	1.38(1.50)	5.62(5.620)	1.61(1.77)	0.39(0.38)
4	817	1.47(1.90)	0.82(0.78)	1.38(1.50)	9.00(8.75)	3.98(4.30)	0.80(0.94)
5	846	1.41(1.53)	0.82(0.82)	1.38(1.50)	15.25(14.00)*	29.23(20.82)#	−0.85(−0.11)
6	442	1.33(1.45)	0.70(0.66)	1.25(1.50)	5.38(5.50)	1.76(1.89)	0.13(0.22)
***P*** **_trend_**		**0.03**					
**Boys**	**1309**	**1.35(1.49)**	**0.71(0.68)**	**1.25(1.25)**	**6.00(6.12)**	**2.01(2.11)**	**0.49(0.56)**
3	201	1.34(1.49)	0.72(0.66)	1.38(1.50)	4.75(4.75)	1.00(1.30)	0.05(−0.06)
4	433	1.46(1.59)	0.78(0.74)	1.38(1.50)	5.25(5.12)	2.41(2.63)	0.95(1.09)
5	437	1.33(1.46)	0.64(0.64)	1.25(1.50)	3.88(4.12)	0.39(0.51)	0.26(0.32)
6	238	1.22(1.34)	0.66(0.63)	1.25(1.25)	4.75(5.12)	1.93(2.02)	−0.33(−0.20)
***P*** **_trend_**		***<0.001***					
**Girls**	**1169**	**1.49(1.61)**	**0.87(0.83)**	**1.50(1.50)**	**15.25(14.0)** *	**18.90(14.73)** #	−**0.30(0.20)**
3	172	1.56(1.67)	0.78(0.76)	1.50(1.63)	5.38(5.12)	1.89(1.75)	0.66(0.65)
4	384	1.48(1.62)	0.87(0.83)	1.50(1.50)	9.00(8.75)	5.08(5.43)	0.66(0.80)
5	409	1.49(1.60)	0.98(0.98)	1.50(1.50)	15.25(14.00)*	30.69(22.80)#	−1.28(−0.37)
6	204	1.47(1.58)	0.71(0.66)	1.50(1.50)	4.50(4.25)	1.25(1.45)	0.50(0.61)
***P*** **_trend_**		***0.76***					

Data in parentheses were determined by retinoscopy.

* There is one outlier with myopia of −8.50 D in the right eye (female, aged 5). When this outlier is excluded, the range decreases to 9.62(10.12), 7.25(8.00), 9.62(10.12) and 7.12(7.88) for all children, 5-year-old children, all girls and 5-year-old girls, respectively.

# When this outlier is excluded, the kurtosis decreases to be 4.59(6.26), 7.55(10.71), 5.79(7.94) and 8.61(11.65) for that in all children, the 5-year-old children, all girls and the 5-year-old girls, respectively.

**Figure 2:**
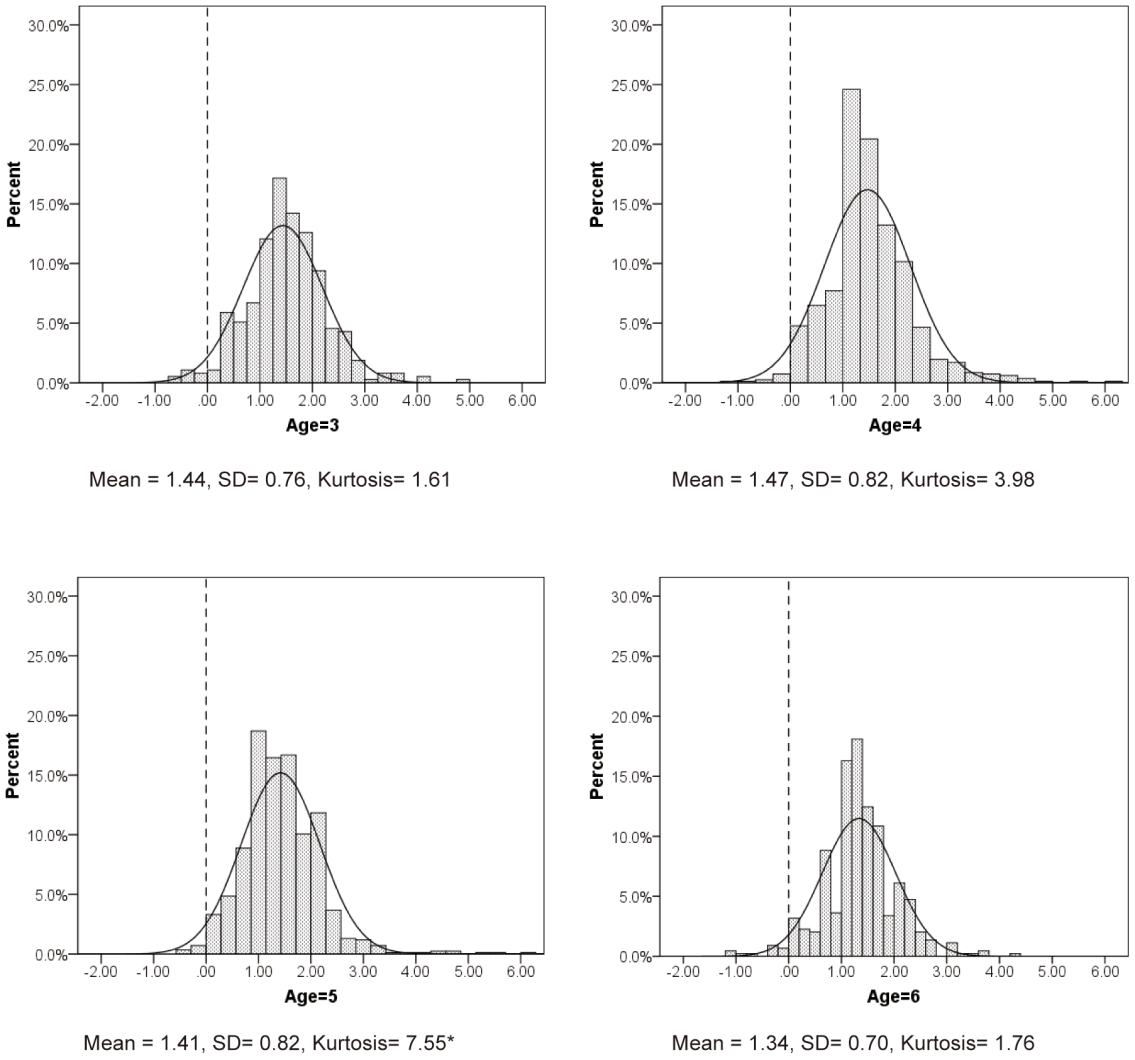
Distributions of spherical equivalent refractive error by age. The rectanglesrepresent the actual distributions of spherical equivalent refractive errors. The red lines represent normal distributions with the same means and standard deviations for each age. *In the group aged 5, the data of one outlier (female, −8,50 D in the right eyes) is not included.

The distribution of refractive errors for each age group is shown in Figure 2. For comparison, normal distributions with the same means and standard deviations are also shown. In all cases, it is clear graphically that the actual distribution of refractive error is tighter than that of the corresponding normal distribution. This is seen most clearly in the increased height of the central peak. Statistical kurtosis is often used to quantify the central tendency in data, but with the exception of one case (one female with −8.50 D in the right eye), the calculated kurtosis is only moderate in our data (from 29.23 to 7.55). In fact, statistical kurtosis is very sensitive to the presence of outliers, and we believe that the graphical evidence of a tighter than normal distribution is more robust than numerical analysis of kurtosis.

### Prevalence of Myopia and Hyperopia

Myopia (SE at least −0.50 D in the worse eye) was uncommon in this paediatric preschool population (Table 3). Using this definition, the overall prevalence of myopia was 1.0% with cycloplegic autorefraction. Significantly different prevalence rates with age were found in all the children (*P* = 0.01) and in the boys (*P* = 0.02), but there was no clear trend in girls. For example, the prevalence of myopia in the boys was 2.5% in year 3, decreased to 0.5% and 0.2% in year 4 and 5, respectively, and then increased again to 1.7% in year 6. This pattern was not observed in the girls (*P* = 0.26). The overall prevalence of myopia in the boys was similar to that in the girls (*P* = 0.78) at all ages (all *P*>0.05). High myopia (SE at least −6.00 D in the worse eye) was extremely rare in this study (0.08%). There were only two children with high myopia (a girl aged 5: −8.50 D, −7.88 D in the right and left eyes, respectively; and a boy aged 6,: −7.62 D, −1.12 D in the right and left eyes, respectively). Using a more stringent definition of at least −1.00 D, prevalence values were naturally lower. To facilitate comparison with other studies, the prevalence of myopia using a definition of at least −0.50 D in the right eye is also shown in Table 3.

**Table 3:** Prevalence of Myopia and Hyperopia in 3–6 year-old Chinese Children Determined by Autorefraction.

Age (yrs)	N	Myopia	Myopia	Myopia	Hyperopia
		(SE ≤ −0.50 D in theworse eye)	(SE ≤−1.00 D in theworse eye)	(SE ≤−0.50 D in theright eye)	(SE ≥+2.00 D in theworse eye)
		N;%; 95%CI	N;%; 95%CI	N;%; 95%CI	N;%; 95%CI
**All**	**2478**	**24; 1.0%; [0.58%–1.35%]**	**10; 0.4%; [0.15%–0.65%]**	**13; 0.5%; [0.24%–0.81%]**	**626; 25.2%; [23.55%–26.97%]**
3	373	8; 2.1%; [0.67%–3.61%]	3; 0.8%; [0.00%–1.71%]	3; 0.8%; [0.00%–1.71%]	106; 28.4%; [23.84%–33.00%]
4	817	7; 0.9%; [0.22%–1.49%]	4; 0.5%; [0.01%–0.97%]	4; 0.5%; [0.01%–0.97%]	222; 27.2%; [24.12%–30.22%]
5	846	2; 0.2%; [0.00%–0.56%]	1; 0.1%; [0.00%–0.35%]	2; 0.2%; [0.00%–0.56%]	209; 24.7%; [21.80%–27.61%]
6	442	7; 1.6%; [0.42%–2.75%]	2; 0.5%; [0.00%–1.08%]	4; 0.9%; [0.02%–1.79%]	89; 20.1%; [16.40%–23.87%]
***P*** **_trend_**		**0.01**			**0.02**
**Boys**	**1309**	**12; 0.9%; [0.40%–1.43%]**	**5; 0.4%; [0.05%–0.72%]**	**7; 0.5%; [0.14%–0.93%]**	**289; 22.1%; [19.83%–24.32%]**
3	201	5; 2.5%; [0.33%–4.64%]	2; 1.0%; [0.00%–2.37%]	2; 1.0%; [0.00%–2.37%]	46; 22.9%; [17.08%–28.69%]
4	433	2; 0.5%; [0.00%–1.10%]	1; 0.2%; [0.00%–0.68%]	0; 0.0%; [0.00%–0.00%]	114; 26.3%; [22.18%–30.48%]
5	437	1; 0.2%; [0.00%–0.68%]	0; 0.0%; [0.00%–0.00%]	1; 0.2%; [0.00%–0.68%]	88; 20.1%; [16.38%–23.90%]
6	238	4; 1.7%; [0.05%–3.31%]	2; 0.8%; [0.00%–2.00%]	4; 1.7%; [0.05%–3.31%]	41; 17.2%; [12.43%–22.02%]
***P*** **_trend_**		**0.02**			**0.03**
**Girls**	**1169**	**12; 1.0%; [0.45%–1.60%]**	**5; 0.4%; [0.05%–0.80%]**	**6; 0.5%; [0.10%–0.92%]**	**337; 28.8%; [26.23%–31.42%]**
3	172	3; 1.7%; [0.00%–3.70%]	1; 0.6%; [0.00%–1.72%]	1; 0.6%; [0.00%–1.72%]	60; 34.9%; [27.76%–42.01%]
4	384	5; 1.3%; [0.17%–2.44%]	3; 0.8%; [0.00%–1.66%]	4; 1.0%; [0.03%–2.06%]	108; 28.1%; [23.63%–32.62%]
5	409	1; 0.2%; [0.00%–0.72%]	1; 0.2%; [0.00%–0.72%]	1; 0.2%; [0.00%–0.72%]	121; 29.6%; [25.16%–34.01%]
6	204	3; 1.5%; [0.00%–3.12%]	0; 0.0%; [0.00%–0.00%]	0; 0.0%; [0.00%–0.01%]	48; 23.5%; [17.71%–29.35%]
***P*** **_trend_**		**0.26**			**0.11**

The overall prevalence of hyperopia (SE at least +2.00 D in the worse eye) was 25.2% in children aged 3 to 6 years (Table 3). The overall prevalence of hyperopia decreased with age, from 28.4% in children aged 3 to 20.1% in those aged 6 (*P* = 0.02). Although significantly different prevalence rates with age were also found in the boys (*P* = 0.03), there was no clear trend. The overall prevalence of hyperopia in the girls was higher than in the boys (*P*<0.001). When age groups were analyzed separately by gender, this trend was observed in the three year-olds and five year-olds *(P* = 0.01 and *P*<0.001, respectively), but not in the 4 and 6 year-olds *(P* = 0.57 and *P* = 0.10, respectively).

**Figure 3:**
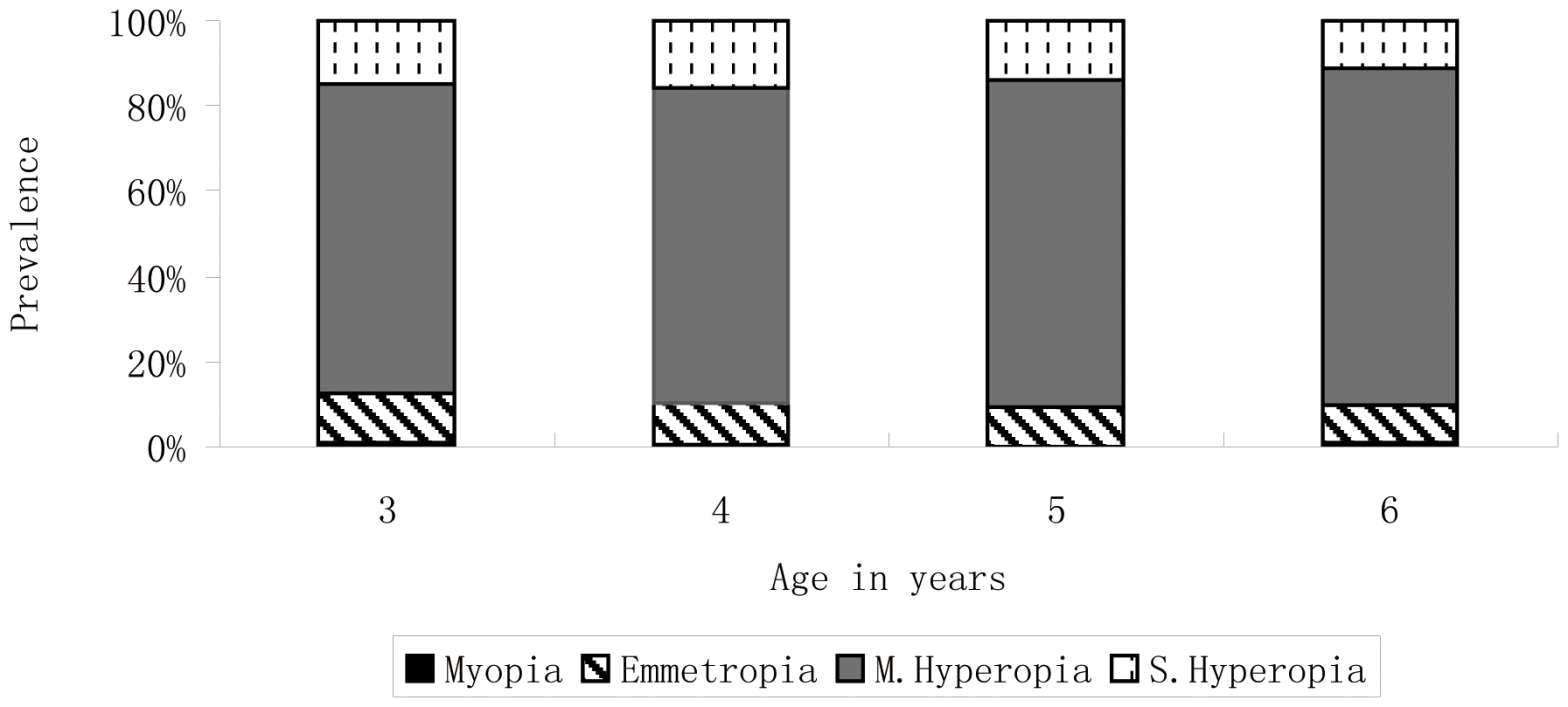
Prevalence of refractive categories by age. To facilitate comparison, definitions identical to those used in a comprehensive analysis of the distributions of refraction error from all the RESC studies [27]are used in this figure. Myopia: </ = −0.5 D, Emmetropia: >−0.5 to </ = +0.5 D, Mild Hyperopia (M. Hyperopia): >+0.5 to </ = +2.0 D, Significant Hyperopia (S. Hyperopia):>+2.0 D.

The distribution of refractive errors is also illustrated graphically in Figure 3. Again, to facilitate comparison, definitions identical to those used in a comprehensive analysis of the distributions of refractive error from all the RESC studies [27] are used in this figure. There was very little myopia detected at any age, and no significant change in the distributions with age. These figures can be directly aligned with those reported using RESC data [27] from slightly older children.

### Prevalence of Astigmatism

Table 4 shows that the mean cylinder power in the right eyes measured by autorefraction was −0.47 D (SD: 0.52) in all the children, and very similar (−0.47 D (SD: 0.53) and −0.48 D (SD: 0.52)) in boys and girls (Table 4). A small but significant age effect was found in all the children (*P* = 0.05), but the trend was not significant when the data was stratified by gender (boys *P* = 0.06, girls *P* = 0.24).

**Table 4:** Mean Cylinder Power and Prevalence of Astigmatism in the Worse Eye Determined by Autorefraction.

Age(yrs)	N	MeanCylinder(D)	SD	Prevalence of astigmatism (Cylinder ≤−1.5 D in the worse eye)			
				N; %; 95%CI	With-the-rule	Against-the-rule	Oblique
					N; %; 95%CI	N; %; 95%CI	N; %; 95%CI
**All**	**2478**	−**0.47**	**0.52**	**202; 8.2%; [7.07%–9.23%]**	**176; 87.1%; [82.51%–91.75%]**	**5; 2.5%; [0.33%–4.62%]**	**21; 10.4%; [6.19%–14.61%]**
3	373	−0.53	0.55	38; 10.2%; [7.12% –13.26%]	32; 84.2%; [72.62%–95.80%]	1; 2.6%; [0.00%–7.72%]	5; 13.2%;[2.41%–23.91%]
4	817	−0.49	0.54	76; 9.3%; [7.31%–11.29%]	67; 88.2%; [80.89%–95.42%]	4; 5.3%; [0.24%–10.28%]	5; 6.6%; [1.01%–12.15%]
5	846	−0.46	0.51	58; 6.9%; [5.15%–8.56%]	48; 82.8%; [73.04%–92.48%]	0; 0.0%; [0.00%–0.00%]	10; 17.2%; [7.52%–26.96%]
6	442	−0.43	0.50	30; 6.8%; [4.44%–9.13%]	29; 96.7%; [90.24%–100.00%]	0; 0.0%; [0.00%–0.00%]	1; 3.3%; [0.00%–9.76%]
***P*** **_trend_**		**0.05**		**0.09**			
**Boys**	**1309**	−**0.47**	**0.53**	**109; 8.3%; [6.83%–9.82%]**	**94; 86.2%; [79.77%–92.71%]**	**3; 2.8%; [0.00%–5.82%]**	**12; 11.0%; [5.13%–16.89%]**
3	201	−0.56	0.59	23; 11.1%; [7.04%–15.84%]	17; 73.9%; [55.97%–91.86%];	1; 4.3%; [0.00%–12.68%]	5; 21.7%; [4.88%–38.60%]
4	433	−0.47	0.50	37; 8.5%; [5.91%–11.18%]	33; 89.2%; [79.18%–99.19%]	2; 5.4%; [0.00%–12.69%]	2; 5.4%; [0.00%–12.69%]
5	437	−0.44	0.55	28; 6.4%; [4.11%–8.70%]	24; 85.7%; [72.75%–98.68%]	0; 0.0%; [0.00%–0.00%]	4; 14.3%; [1.32%–27.25%]
6	238	−0.45	0.49	21; 8.8%; [5.22%–12.43%]	20; 95.2%; [86.13%–100.00%]	0; 0.0%; [0.00%–0.00%]	1; 4.8%; [0.00%–13.87%]
***P*** **_trend_**		**0.06**		**0.19**			
**Girls**	**1169**	−**0.48**	**0.52**	**93; 8.0%; [6.40%–9.51%]**	**82; 88.2%; [81.61%–94.74%]**	**2; 2.2%; [0.00%–5.10%]**	**9; 9.7%; [3.67%–15.69%]**
3	172	−0.48	0.49	15; 8.7%; [4.50%–12.94%]	15; 100.0%; [100.00%–100.00%]	0; 0.0%; [0.00%–0.00%]	0; 0.0%; [0.00%–0.00%]
4	384	−0.51	0.58	39; 10.2%; [7.13%–13.18%]	34; 87.2%; [76.69%–97.67%]	2; 5.1%; [0.00%–12.05%]	3; 7.7%; [0.00%–16.06%]
5	409	−0.48	0.52	30; 7.3%; [4.81%–9.86%]	24; 80.0%; [65.69%–94.31%]	0; 0.0%; [0.00%–0.00%]	6; 20.0%; [5.69%–34.31%]
6	204	−0.41	0.44	9; 4.4%; [1.59%–7.23%]	9; 100.0%; [100.00%–100.00%]	0; 0.0%; [0.00%–0.00%]	0; 0.0%; [0.00%–0.00%]
***P*** **_trend_**		**0.24**		**0.09**			

The overall prevalence of astigmatism (at least 1.50 D in the worse eye) was 8.2% (Table 4). There was no statistically significant age effect on the prevalence of astigmatism for all children (*P* = 0.09), boys (*P* = 0.19) and girls (*P* = 0.09). The overall prevalence of astigmatism was similar in boys (8.3%) and girls (8.0%) (*P* = 0.74). No significant difference in the prevalence of astigmatism was found between the boys and girls across all the ages (all *P*>0.05). With-the-rule astigmatism was overwhelmingly the most common type of astigmatism, followed by oblique and against-the-rule type, at all ages in both boys and girls (Table 4).

## Discussion

The results obtained on this sample of pre-school children from Guangzhou in this study differ markedly from those reported for Chinese children in the Singapore STARS study [20]. In Guangzhou, the prevalence of myopia was very low for the ages 3–6 years, and the mean SE was more hyperopic than found in Singapore. We have not studied children under the age of 3, and our results therefore do not rule out higher prevalences of myopia at younger ages. In contrast to the differences in spherical equivalent refraction, the prevalence of astigmatism (cylinder at least 1.50 D) appeared to be similar in the two studies, and where the age ranges over-lapped, the prevalence of astigmatism was stable with age. At earlier ages, there appeared to be a declining trend in the prevalence of astigmatism.

There was very little myopia detected over the age range studied, and, apart from some decline in the prevalence of hyperopia, little change in refractive error. The results that we have obtained are therefore consistent with the conventional pattern of refractive development based on studies of white children in Europe and North America [1]–[6]. These results suggest that the prevalence of myopia may have been over-estimated in STARS, and the prevalence of hyperopia may have been underestimated. The latter parameter is particularly sensitive to inadequate cycloplegia. These two errors would then combine to produce more myopic/less hyperopic estimates of mean SE.

It could be argued that the prevalence of myopia is likely to be particularly high in Chinese children in Singapore, given the high prevalence of close to 30% reported for 5–6 year-old children in the SCORM study [28], and given that this high prevalence of myopia might be detected earlier in development. However, the prevalence of myopia in the SCORM study is significantly higher than the prevalence of myopia reported for children of the same age in the STARS study [20], perhaps because the SCORM population was not selected by population-based randomisation, whereas that of the STARS study was. Furthermore, older Chinese children in Guangzhou are now highly myopic, with the prevalence of myopia ranging from 3.3% (retinoscopy) and 5.7% (autorefraction) in 5 year-olds, up to 73.1% (retinoscopy) and 78.4% (autorefraction) in 15 year-olds [11]. Thus, the expectation would be for similar prevalences in preschool children in Singapore and Guangzhou.

One of the features of the MEPEDS, BPEDS and STARS studies is a significant level of myopia in the youngest age group studied, in all ethnic groups other than white. We have not studied children under the age of 3, and there are few studies on such children of Chinese origin. However, Thorn and colleagues [29] have examined Chinese neonates, finding that the mean SE was +3.55 D, while the mean SE without cycloplegia was +0.58 D. The major difference between cycloplegic and non-cycloplegic refraction illustrates the importance of adequate cycloplegia in young children. The results of Thorn and colleagues suggest that Chinese neonates are highly hyperopic, as are neonates of European origin [30], [31]. This conclusion is also supported by the results of Chan and Edwards [32]. In carrying out studies on pediatric populations, we therefore believe that particular attention needs to be devoted to methodological issues, of which the first is the use of a standard method for refraction for all ages. To cover the age range from birth until 5–6 years of age, the two choices are currently the Retinomax hand-held autorefractor and retinoscopy. We found no difference between the results of autorefraction with a table-mounted autorefractor and retinoscopy over the more limited age range we have studied, but there are age limits to the use of table-mounted autorefactors. Given the evidence that the Retinomax autorefractor overestimates myopia, we suggest that retinoscopy is the method of choice.

The second issue is that of adequate cycloplegia. STARS, as well as BPEDS, MEPEDS used two drops of 0.5% cycloplegia in the 6–12 months groups. While the reasons for using milder cycloplegia in younger children can be readily appreciated, this may lead to overestimation of myopia in the youngest children studied, and may account for the apparent decreases in the prevalence of myopia with age. Thorn and colleagues used 2 drops of a mixture of 0.5% cyclopentolate and 2.5% phenylephrine, administered 10 minutes apart in neonates, and found much more hyperopia and much less myopia. The apparent effectiveness of cycloplegia using this regime in neonates is perhaps surprising, but Chan and Edwards [32] have reported a rapid decline in the effectiveness of cycloplegia over the first few months after birth.

In older children, MEPEDS and BPEDS used two drops of 1% cyclopentolate, without explicit criteria for assessment of effective cycloplegia. STARS used three drops of 1% cyclopentolate, without explicit criteria for adequacy, while the Guangzhou RESC study used three drops of 1% cyclopentolate as a standard, with rigorous assessment of cycloplegia through criteria for dilation and light reflex. Data where cycloplegia was deemed to be incomplete were excluded, which occurred in approximately 10% of cases, largely due to inadequate dilation. Our protocol was to use 3 drops of 1% cyclopentolate as a standard, and to use an additional drop of cyclopentolate if a light reflex was still visible. In 8–10% of cases, an additional drop was administered. No children required more than 4 drops for cycloplegia to be judged adequate. For assessing the effectiveness of cycloplegia, systematic use of retinoscopy provides a considerable advantage, since changes in pupil size in response to light are readily detectable with this technique.

There is clearly some parallel between the stringency of the cycloplegia regimes used, and the reported prevalences of myopia. The protocol we have used of up to 4 drops of 1% cyclopentolate may be required to eliminate pseudo-myopia, particularly in children with dark irises, and there is therefore a need for caution in assuming that low prevalences of myopia detected at an early age represent real myopia rather than pseudo-myopia. It should be noted that in about 1% of cases, facial reddening and thirst were reported as side-effects, and thus a careful balance has to be struck between achieving adequate cycloplegia and avoiding systemic side-effects, even though, in this study, no other serious effects were reported.

Our results therefore support the idea that in Chinese children in the age range of 3–6, there is little myopia, and more hyperopia than is often observed, using a rigorous cycloplegic regime. In STARS, using a cut-off of at least −0.50 D, the prevalence of myopia at age 3 was reported as 8.6%, as compared to 0.67% in this study. The comparable values for hyperopia of at least +2.00 D are 5.1% in STARS and 28.4% in this study, and for mean SE are +0.61 D and +1.44 D respectively. A shift towards more myopic refractions, with considerable loss of hyperopia, is a characteristic feature of measurements made without cycloplegia or with inadequate cycloplegia. Overall, this comparison suggests that refractive development in Chinese children over this age range is very similar to that seen in children of European origin, with little myopia detected until children reach the age of 5–6, provided that cycloplegia is adequate. Other results suggest that neonatal refractive development in Chinese children may also be similar to that in children of European origin [29], [32]. After the age of 5–6, possibly due to the impact of high amounts of study and limited time outdoors [33]–[35], the prevalence of myopia increases markedly in some East Asian populations to produce the current myopia epidemic [36]–[38]. We have not studied children of African-American or Hispanic ethnicity, but, if children of Chinese origin follow the conventional pattern, as our results suggest, then we believe that early refractive development needs to be re-examined in children from these, and other, ethnic groups, particularly those with dark irises. Given the low prevalence values reported for myopia in these paediatric studies, only a small proportion of children with less than adequate cycloplegia could account for the prevalence of myopia reported in other studies.

One limitation of our study is that it is preschool-based, while the STARS study used population enumeration and recruitment. However, we do not believe that this is likely to be the reason for the difference in results, given that the level of preschool attendance in Guangzhou is now over 80%, and the participation rate was over 75%. This compared to a slightly lower participation rate in STARS, combined with some selective participation based on proximity to study sites. If we make the assumption that myopia at the rate reported for Chinese children in Singapore is concentrated in those who do not attend preschool in Guangzhou, or who did not participate in the study, the prevalence of myopia would have to be between 10% and 20% in this group of children. This seems unlikely, particularly given that non-attendance and non-participation are more likely to be associated with low SE and low parental education, where children are less likely to be myopic.

In summary, our results call into question the patterns of refractive development reported for Chinese children in the STARS study. By implication, the similar patterns, characterised by significant neonatal myopia which declines in prevalence over the next few years, reported in the MEPEDS and BPEDS studies in children from ethnic backgrounds other than white, are also called into question. Without strong control of cycloplegia, it is difficult to be sure that the low prevalence rates of myopia reported in these studies represent genuine myopia, rather than pseudo-myopia.

The methodological issues concerning cycloplegia we have raised need to be clarified, before definitive conclusions can be reached. But, we believe that much of the current evidence favours the idea that, in Chinese children, the conventional picture of refractive development remains valid. This includes marked neonatal hyperopia, rapid elimination of neonatal myopia and the slower and more progressive elimination of hyperopia, with development of a highly kurtotic distribution of SE, combined with very low prevalence rates for myopia prior to the age of 5–6. At this stage, the idea that there is a common pattern to early refractive development across ethnic groups should not be abandoned. The process of early emmetropisation, leading to a tight distribution of refractive error with a hyperopic mean SE may therefore provide a developmental bottle-neck through which children of all ethnic groups pass up to the age of 5–6, after which differences in environmental exposures such as educational pressures and time outdoors [33]–[35], between ethnic groups and locations, produce the major differences in the prevalence of myopia currently seen internationally [36]–[38]. It also seems possible that in locations or populations where there is very early onset of the myopigenic behavioural pattern of considerable nearwork and limited time outdoors, myopia may begin to appear prior to the age of 5–6. But distinguishing between low levels of myopia and low levels of pseudo-myopia will be difficult, particularly when less powerful cycloplegic regimes are used.
